# Cost-Effectiveness of Neoadjuvant-Adjuvant Treatment Strategies for Women With *ERBB2* (*HER2*)–Positive Breast Cancer

**DOI:** 10.1001/jamanetworkopen.2020.27074

**Published:** 2020-11-23

**Authors:** Natalia Kunst, Shi-Yi Wang, Annette Hood, Sarah S. Mougalian, Michael P. DiGiovanna, Kerin Adelson, Lajos Pusztai

**Affiliations:** 1 Department of Health Management and Health Economics, University of Oslo, Oslo, Norway; 2 Cancer Outcomes, Public Policy, and Effectiveness Research (COPPER) Center, Yale School of Medicine, New Haven, Connecticut; 3 Public Health Modeling Unit, Yale University School of Public Health, New Haven, Connecticut; 4 Department of Epidemiology and Data Science, Amsterdam University Medical Centers, Amsterdam, the Netherlands; 5 Department of Chronic Disease Epidemiology, Yale University School of Public Health, New Haven, Connecticut; 6 Smilow Cancer Hospital, Yale New Haven Hospital, New Haven, Connecticut; 7 Yale Cancer Center, Yale School of Medicine, New Haven, Connecticut

## Abstract

**Importance:**

The neoadjuvant treatment options for *ERBB2*-positive (also known as *HER2*-positive) breast cancer are associated with different rates of pathologic complete response (pCR). The KATHERINE trial showed that adjuvant trastuzumab emtansine (T-DM1) can reduce recurrence in patients with residual disease compared with patients treated with trastuzumab; however, T-DM1 and other *ERBB2*-targeted agents are costly, and understanding the costs and health consequences of various combinations of neoadjuvant followed by adjuvant treatments in the United States is needed.

**Objective:**

To examine the costs and disease outcomes associated with selection of various neoadjuvant followed by adjuvant treatment strategies for patients with *ERBB2*-positive breast cancer.

**Design, Setting, and Participants:**

In this economic evaluation, a decision-analytic model was developed to evaluate various neoadjuvant followed by adjuvant treatment strategies for women with *ERBB2*-positive breast cancer from a health care payer perspective in the United States. The model was informed by the KATHERINE trial, other clinical trials with different regimens from the KATHERINE trial, the Flatiron Health Database, McKesson Corporation data, and other evidence in the published literature. Starting trial median age for KATHERINE patients was 49 years (range, 24-79 years in T-DM1 arm and 23-80 years in trastuzumab arm). The model simulated patients receiving 5 different neoadjuvant followed by adjuvant treatment strategies. Data analyses were performed from March 2019 to August 2020.

**Exposure:**

There were 4 neoadjuvant regimens: (1) HP: trastuzumab (H) plus pertuzumab (P), (2) THP: paclitaxel (T) plus H plus P, (3) DDAC-THP: dose-dense anthracycline/cyclophosphamide (DDAC) plus THP, (4) TCHP: docetaxel (T) plus carboplatin (C) plus HP. All patients with pCR, regardless of neoadjuvant regimen, received adjuvant H. Patients with residual disease received different adjuvant therapies depending on the neoadjuvant regimen according to the 5 following strategies: (1) neoadjuvant DDAC-THP followed by adjuvant H, (2) neoadjuvant DDAC-THP followed by adjuvant T-DM1, (3) neoadjuvant THP followed by adjuvant DDAC plus T-DM1, (4) neoadjuvant HP followed by adjuvant DDAC/THP plus T-DM1, or (5) neoadjuvant TCHP followed by adjuvant T-DM1.

**Main Outcomes and Measures:**

Lifetime costs in 2020 US dollars and quality-adjusted life-years (QALYs) were estimated for each treatment strategy, and incremental cost-effectiveness ratios were estimated. A strategy was classified as dominated if it was associated with fewer QALYs at higher costs than the alternative.

**Results:**

In the base-case analysis, costs ranged from $415 833 (strategy 3) to $518 859 (strategy 4), and QALYs ranged from 9.67 (strategy 1) to 10.73 (strategy 3). Strategy 3 was associated with the highest health benefits (10.73 QALYs) and lowest costs ($415 833) and dominated all other strategies. Probabilistic analysis confirmed that this strategy had the highest probability of cost-effectiveness (>70% at willingness-to-pay thresholds of $0-200,000/QALY) and was associated with the highest net benefit.

**Conclusions and Relevance:**

These results suggest that neoadjuvant THP followed by adjuvant H for patients with pCR or followed by adjuvant DDAC plus T-DM1 for patients with residual disease was associated with the highest health benefits and lowest costs for women with *ERBB2*-positive breast cancer compared with other treatment strategies considered.

## Key Points

**Question:**

What is the cost-effective neoadjuvant-adjuvant treatment strategy among several clinically reasonable alternatives for human *ERBB2*-positive breast cancer in the United States?

**Findings:**

In this economic evaluation, based on the KATHERINE trial and other clinical trials and evidence sources, neoadjuvant trastuzumab plus paclitaxel plus pertuzumab followed by adjuvant trastuzumab for patients with pathologic complete response or followed by adjuvant dose-dense anthracycline/cyclophosphamide and trastuzumab emtansine for patients with residual disease was associated with the highest health benefits and least costs among 5 strategies considered.

**Meaning:**

These findings suggest that, among 5 neoadjuvant-adjuvant treatment strategies, 1 strategy is associated with not only improved health outcomes but also cost savings.

## Teaser

This economic evaluation modeled the cost-effectiveness of 5 neoadjuvant-adjuvant treatment strategies for women with *ERBB2*-positive breast cancer.

## Introduction

Preoperative (ie, neoadjuvant) chemotherapy in combination with *ERBB2* (also known as growth factor receptor 2 [*HER2*])–targeted agents is increasingly used in the treatment of stage II to III *ERBB2*-positive breast cancer because this treatment strategy can lead to increased breast conservation and smaller resection volumes^1^ and the extent of residual cancer can guide subsequent postoperative (ie, adjuvant) treatment.^2^ Patients with pathologic complete response (pCR), defined as no residual invasive cancer in the breast or lymph nodes (ie, ypT0/is and ypN0), have excellent overall survival rates, regardless of neoadjuvant chemotherapy regimen used.^3,4^ Rates of pCR range from 6% to 80% in *ERBB2*-positive breast cancer, depending on regimen and estrogen receptor (ER) status. Trastuzumab and pertuzumab without any chemotherapy can result in 6% pCR rate in ER-positive/*ERBB2*-positive cancers,^5^ whereas the combination of trastuzumab and pertuzumab with sequential anthracycline and taxane chemotherapy can result in pCR rates as high as 80% in ER-negative/*ERBB2*-positive cancers.^6,7^ Equally importantly, the decreased survival rates of patients with residual *ERBB2*-positive disease compared with individuals with pCR can be improved by additional, adjuvant therapy with trastuzumab emtansine (T-DM1). The KATHERINE trial^8^ compared adjuvant T-DM1 with adjuvant trastuzumab in patients who had residual disease after neoadjuvant chemotherapy and *ERBB2*-targeted therapy. Of 1486 patients included in the trial (743 individuals in the T-DM1 arm and 743 individuals in the trastuzumab arm), all received a taxane, 1143 individuals (77%) received an anthracycline in the neoadjuvant setting, and 290 individuals (20%) received dual *ERBB2*-targeted therapy (eg, trastuzumab plus pertuzumab) concurrent with chemotherapy. The trial showed significantly improved invasive disease–free survival (hazard ratio [HR], 0.50; 95% CI, 0.39-0.64) and distant metastasis–free survival (HR, 0.60; 95% CI, 0.45-0.79) with T-DM1.^8^ In a similar trial in individuals with *ERBB2*-negative breast cancer, adjuvant capecitabine improved disease-free and overall survivals in patients with residual disease after neoadjuvant chemotherapy that contained anthracycline, taxane, or both.^9^ These randomized clinical trials in different disease subtypes demonstrated the clinical principle that further adjuvant chemotherapy for patients with residual disease after neoadjuvant chemotherapy can improve outcome.

For *ERBB2*-positive breast cancer, there are several neoadjuvant chemotherapy options, each associated with different costs, toxic effects, and rates of pCR. This study examined the cost-effectiveness of different neoadjuvant-adjuvant treatment strategies in the United States. We assume that (1) breaking up a sequential, multidrug regimen into preoperative and postoperative components will result in the same overall outcome as administering all treatment preoperatively,^10^ (2) patients who achieve pCR will have similarly good prognosis regardless of what regimen, or regimen component, induced pCR,^4,11^ and (3) patients with the same residual disease amount have similar prognosis, regardless of type of neoadjuvant regimen. We based our decision-analytic model on the KATHERINE trial population and outcome and on data from other clinical trials for regimens that were not used in that trial.

## Methods

### Decision-Analytic Model

**Figure 1.**
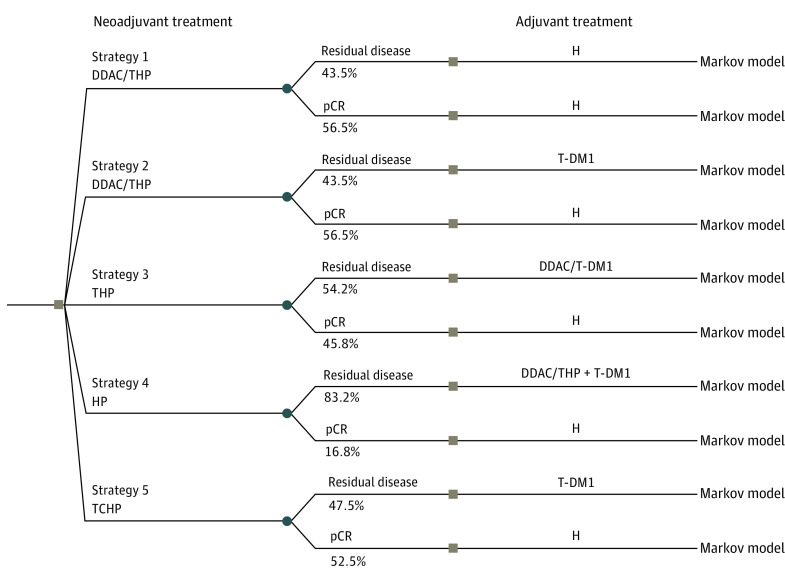
Structure of Decision Tree Squares indicate decision nodes; circles, event (ie, chance) nodes; DDAC, dose dense anthracycline/cyclophosphamide; DDAC/THP, DDAC followed by paclitaxel, trastuzumab, and pertuzumab; H, trastuzumab; HP, trastuzumab and pertuzumab; pCR, pathologic complete response; TCHP, docetaxel, carboplatin, trastuzumab, and pertuzumab; T-DM1, trastuzumab emtansine; and THP, paclitaxel, trastuzumab, and pertuzumab triplet.

This economic evaluation used no individual patient-level data to inform the decision-analytic model. Therefore, it does not constitute human participant research and does not require institutional review board review or exemption according to US Department of Health and Human Services 45 CFR part 46. Our decision-analytic model comprised a decision tree and a state-transition Markov model, developed following the Consolidated Health Economic Evaluation Reporting Standards (CHEERS) reporting guideline^12^ and using R statistical software version 3.6.2 (R Project for Statistical Computing).^13^ The decision tree included 5 different neoadjuvant-adjuvant treatment strategies and distributed patients into 1 of the Markov models (Figure 1). The simulated study population was modeled after the KATHERINE trial,^8^ which included 1486 patients with a starting trial median age of 49 years (range, 24-79 years in T-DM1 arm and 23-80 years in trastuzumab arm) in US settings. The model considered 4 neoadjuvant regimens: (1) HP: trastuzumab (H) and pertuzumab (P); (2) THP: paclitaxel (T), H, and P; (3) DDAC/THP: dose-dense anthracycline/cyclophosphamide (DDAC) followed by THP; and (4) TCHP: docetaxel, carboplatin, H, and P. Patients with pCR after any of these neoadjuvant regimens received H in the adjuvant setting. Patients with residual disease received adjuvant therapies depending on their prior neoadjuvant therapy, resulting in 5 overall neoadjuvant-adjuvant treatment strategies: (1) neoadjuvant DDAC/THP followed by adjuvant H for patients with pCR or residual disease, (2) neoadjuvant DDAC/THP followed by adjuvant T-DM1 for patients with residual disease and followed by adjuvant H for patients with pathological CR, (3) neoadjuvant THP followed by adjuvant DDAC followed by T-DM1 for patients with residual disease (similar to the KATHERINE neoadjuvant regimen but split into preoperative and postoperative components) and followed by adjuvant H for patients with pathological CR, (4) neoadjuvant HP (a nonchemotherapy neoadjuvant regimen) followed by adjuvant DDAC/THP plus T-DM1 for patients with residual disease and followed by adjuvant H for patients with pathological CR, and (5) neoadjuvant TCHP followed by adjuvant T-DM1 for patients with residual disease and followed by adjuvant H for patients with pathological complete response (Figure 1).

Strategies 3 and 4 have not been tested in clinical trials, but they represent logical extensions of our underlying assumptions about the pCR prognostic function independent of what regimen has induced this response and about the equal efficacy of a multidrug regimen when it is administered as neoadjuvant therapy or broken up into neoadjuvant and adjuvant components. We also point out that strategy 3 is going to be tested in the CompassHER2-pCR trial (ECOG-ACRIN EA1181; NCT04266249).^14^

### Model Structure

**Figure 2.**
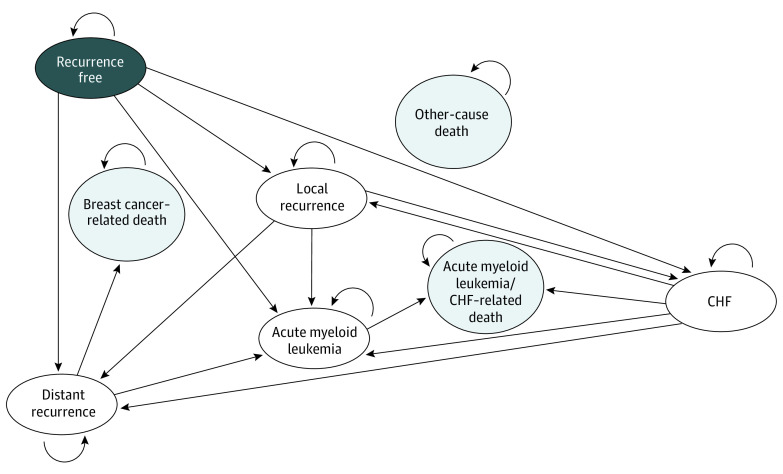
Structure of State-Transition Markov Model All patients started in the recurrence-free health state and were at risk of developing local recurrence or distant recurrence. In strategies 1-4, all patients who received chemotherapy were also at risk of developing acute myeloid leukemia or congestive heart failure (CHF), health states that represent long-term toxic effects from chemotherapy. The model has 4 absorbing health states for breast cancer–related, acute myeloid leukemia–related, CHF-related, and age-dependent other-cause death. The 2 absorbing health states, acute myeloid leukemia–related death and CHF-related death, are presented together.

The Markov model with 4 main health states (ie, recurrence free, local recurrence, distant recurrence, and death) simulated lifetime costs and quality-adjusted life-years (QALYs) associated with neoadjuvant-adjuvant regimen combinations, applying a 3% discounting rate (Figure 2).^15^ The model also accounted for chemotherapy toxic effects with 2 additional health states: acute myeloid leukemia and congestive heart failure (CHF). Death state included breast cancer–related, acute myeloid leukemia–related, CHF-related, and age-dependent other-cause death.

### Clinical Parameters

We performed scoping literature searches to identify the best available evidence for clinical parameters. Proportions of patients achieving pCR with different neoadjuvant treatments were taken from clinical trials^4,5,6,7,8,16,17,18,19,20,21,22,23,24,25,26,27,28,29,30,31,32,33,34,35,36,37,38^ and were 16.8% for HP, 45.8% for THP, 56.5% for DDAC/THP, and 52.5% for TCHP (Table 1). We simulated a population similar to that in the KATHERINE trial and used recurrence estimates from the study.^8^ For patients with residual disease treated with adjuvant H, we assumed a 3-year probability of distant recurrence of 15.9%.^8^ For other adjuvant treatments with residual disease, we used the following relative risks (RR) of distant recurrence: T-DM1: 0.60; DDAC/THP followed by T-DM1: 0.52; and DDAC followed by T-DM1: 0.40^4,8^ (Table 1). For patients with pCR in all strategies, we assumed a 5-year probability of distant recurrence of 5% (ie, RR of distant recurrence, 0.18)^4^ (Table 1). Local recurrence risks after pCR or residual disease were taken from a 2017 publication^4^ and the KATHERINE trial,^8^ but we also postulated that patients with pCR could not have a higher locoregional recurrence risk compared with patients with residual disease.

**Table 1.** Input Parameters for Decision-Analytic Model

Input parameters	Value	Probability distribution^a^
Proportion of patients with pCR after neoadjuvant treatment, %		
HP	16.8	β (α = 18.00; β = 89.00)
THP	45.8	β (α = 49.00; β = 58.00)
DDAC/THP	56.5	β (α = 78.00; β = 60.00)
TCHP	52.5^b^	β (α = 115.00; β = 104.00)
**Effect of adjuvant treatment**		
Distant recurrence^c^		
3-y distant recurrence probability with H with residual disease (reference group), %	15.9	β (α = 118.00; β = 625.00)
RR by adjuvant treatment		
T-DM1 with residual disease	0.60	Log normal (μ = −0.51; σ = 0.09)
DDAC/THP followed by T-DM1 with residual disease	0.52^d^	Truncated normal (a = 0.18; b = 0.60)^e^
DDAC followed by T-DM1 with residual disease	0.40^d^	Truncated normal (a = 0.18; b = 0.60)^e^
H with pCR	0.18	Log normal (μ = −1.70; σ = 0.18)
Local recurrence^c^		
3-y locoregional recurrence probability for H with residual disease (reference group), %	4.6	β (α = 34.00; β = 709.00)
RR by adjuvant treatment		
All treatments with residual disease other than H	0.24^f^	Log normal (μ = −1.43; σ = 0.11)
H with pCR	0.24^g^	Log normal (μ = −1.43, σ = 0.11)
Subsequent distant recurrence after initial local recurrence		
10-y probability, %	18.9^h^	β (α = 13.00; β = 56.00)
Survival and mortality parameters		
Median survival, mo		
With distant recurrence	38	Normal (38.00; 4.08)
With acute myeloid leukemia	8	Normal (8.00; 2.00)
Mortality recurrence-free state	Background mortality, life table, age-dependent	NA
Annual risk of death due to CHF, %	12.7%	β (α = 69.93; β = 488.07)
**Chemotherapy toxicity**^c^		
CHF		
1-y probability in patients with non-AC chemotherapy (reference group), %	3.7	β (α = 100.32; β = 2647.72)
RR for AC chemotherapy	1.26	Log normal (μ = 0.23; σ = 0.08)
Acute myeloid leukemia		
1-y probability in patients with no chemotherapy (reference group), %	0.1%	β (α = 138.30; β = 197 505.60)
RR for non-AC chemotherapy	0.88	Log normal (μ = −0.13; σ = 0.35)
RR for AC chemotherapy	1.68	Log normal (μ = 0.52; σ = 0.28)
**Costs, $**^i^		
Neoadjuvant treatment regimen^j^		
HP	64 389	γ (α = 25.00; β = 2575.56)
THP	65 428	γ (α = 25.00; β = 2617.10)
DDAC/THP	106 787	γ (α = 25.00; β = 4271.49)
TCHP	153 257	γ (α = 25.00; β = 6130.28)
Adjuvant treatment regimen^j^		
H	108 995	γ (α = 25.00; β = 4359.78)
T-DM1	157 871	γ (α = 25.00; β = 6314.82)
DDAC/THP followed by T-DM1	264 658	γ (α = 25.00; β = 10586.32)
DDAC followed by T-DM1	199 230	γ (α = 25.00; β = 7969.21)
Adjuvant H after neoadjuvant TCHP	93 424	γ (α = 25.00; β = 3736.96)
Adjuvant T-DM1 after neoadjuvant TCHP	135 318	γ (α = 25.00; β = 5412.70)
**Treatment cost of recurrence, $**		
Locoregional recurrence		
First y	21 005^k^	γ (α = 25.00; β = 840.20)
After first y	2335^k^	γ (α = 25.00; β = 93.41)
Distant recurrence		
Annual cost of care	144 865^l^	γ (α = 25.00; β = 5794.62)
Chemotherapy toxic effects		
Initial CHF treatment	36 748	γ (α = 25.00; β = 1469.92)
Annual CHF care	7035	γ (α = 25.00; β = 281.40)
Lifetime treatment of acute myeloid leukemia	21 345	γ (α = 2530.10; β = 1/8.44)
Utilities of health states		
First y recurrence free	0.79	β (α = 87.73; β = 24.17)
Second y and after		
Without recurrence	0.83	β (α = 39.01; β = 8.33)
With local recurrence	0.72	β (α = 89.85; β = 34.60)
With distant recurrence	0.53	β (α = 4.61; β = 4.13)
With CHF	0.71	β (α = 72.38; β = 29.57)
With acute myeloid leukemia	0.26	β (α = 9.13; β = 25.98)
Last y with distant recurrence before death	0.16	β (α = 5.00; β = 26.26)

Abbreviations: CHF, congestive heart failure; DDAC, dose-dense anthracycline/cyclophosphamide; DDAC/THP, dose-dense anthracycline/cyclophosphamide followed by paclitaxel, trastuzumab, and pertuzumab; H, trastuzumab; HP, trastuzumab and pertuzumab; NA, not applicable; pCR, pathologic complete response; RR, relative risk; TCHP, docetaxel, carboplatin, trastuzumab, and pertuzumab; T-DM1, trastuzumab emtansine; THP, paclitaxel, trastuzumab, and pertuzumab triplet.

^a^ Probability distributions of clinical and utility parameters were informed with summary statistics. For most cost parameters, no summary statistics were available, and we therefore assumed a 20% SE.

^b^ This estimate was obtained using estimates for estrogen receptor–positive cancer and estrogen receptor–negative cancer and the proportion of patients with each type in the KATHERINE trial.

^c^ We converted risks of recurrence, acute myeloid leukemia, and CHF to 1-year probabilities and used these in the model in the form of RRs.

^d^ This is an assumption because of a lack of data for this setting. We assumed that the true value was between a 5-year probability of distant recurrence of 5% in patients with pCR receiving H (from Symmans et al^4^) for the proportion of patients who would have achieved pCR if treated with neoadjuvant DDAC/THP and a 3-year probability of distant recurrence for patients with residual disease receiving T-DM1 (from von Minckwitz et al^8^).

^e^ A log-normal distribution was also examined for RR of distant recurrence for adjuvant DDAC/THP followed by T-DM1 with residual disease and RR of distant recurrence for adjuvant DDAC followed by T-DM1 with residual disease. We found that applying the log-normal distribution to these parameters did not alter the cost-effectiveness results of our study, and we assumed that the truncated normal distribution would better reflect assumptions of our study and characterize uncertainty in these parameters.

^f^ There is no data on probability of local recurrence in patients with residual disease receiving DDAC/THP followed by T-DM1 or DDAC followed by T-DM1. Thus, we made a conservative assumption that it was equal to T-DM1 alone.

^g^ Patients with pCR receiving H have a better prognosis than patients with residual disease receiving H. Thus, the local recurrence probability in the group H with pCR cannot be higher than the local recurrence probability in the group receiving H with residual disease. Gianni et al^5^ reported higher local recurrence probabilities for patients with pCR because that study enrolled a higher-risk population at baseline than the KATHERINE trial. Consequently, we based the estimates of the local-recurrence probabilities for patients receiving H with pCR on the KATHERINE trial and assumed that these estimates were the same as estimates for the group receiving H with residual disease.

^h^ The estimate was calculated using the number of patients who developed subsequent distant recurrence after an initial local recurrence during a 10-year period of the study by Wapnir et al.^34^

^i^ All costs are expressed in 2020 US dollars. When necessary, we inflated unit costs to 2020 US dollars using the Consumer Price Index.

^j^ We used drug-pricing data from McKesson Corporation to calculate the costs of each treatment regimen.

^k^ A mean of local and regional recurrence provided by Schousboe et al^29^ and inflated with Consumer Price Index from January 2008 to January 2020.

^l^ The cost of distant-recurrence health state was estimated using the Flatiron Health Database for use of treatment regimens among patients with metastatic breast cancer and drug-pricing data from McKesson Corporation. We used utilization data for patients diagnosed after the Food and Drug Administration approval of T-DM1 (ie, March 2017 to July 2019).

We recognize that treatment strategies 3 and 4 have not been formally tested in clinical trials, to our knowledge, but we assumed that overall outcomes in the residual disease cohorts of these regimens would be similar to those seen in the KATHERINE experimental arm, because the total chemotherapy received is the same. In this analysis, we did not consider adjuvant endocrine therapy, because we assumed that patients with ER-positive cancer would be represented equally and treated uniformly with adjuvant endocrine therapy in each of the 5 treatment strategies. For patients receiving chemotherapy, we also accounted for the probability, according to treatment regimen, of experiencing CHF or acute myeloid leukemia using estimates from the published literature.^18,19^ Input parameters are provided with probability distributions (Table 1; eTable 1 in the Supplement).

### Quality of Life and Costs

We estimated patients’ quality of life by assigning different utility weights to each health state taken from published literature (Table 1).^20,21,22,23,24,25,26,27^ We used drug-pricing data from McKesson Corporation^28^ to calculate the costs of each treatment regimen (eTable 1 in the Supplement). For local recurrence, acute myeloid leukemia, and CHF health states, we took cost estimates from the published literature (Table 1; eTable 1 in the Supplement).^25,29,30,31,32^ We estimated the costs of distant recurrence health state using the Flatiron Health Database for different treatment regimen utilization among patients with *ERBB2*-positive metastatic breast cancer from 2017 to 2019, using McKesson^28^ data for drug prices. All costs were expressed in 2020 US dollars, adjusted with the consumer price index (CPI).^33^

### Statistical Analysis

#### Base-Case Analysis

The main outcomes of the decision-analytic model were costs and QALYs associated with each treatment strategy. We first ranked the 5 strategies by their costs, then estimated incremental costs and incremental QALYs, and then calculated incremental cost-effectiveness ratios (ICERs). ICER represents incremental costs per QALY gained relative to the next least-costly strategy. We classified a strategy as *dominated* if it was associated with fewer QALYs at higher costs than the alternative. We classified a strategy as *cost-effective* if it was associated with the highest ICER below the willingness-to-pay threshold considered. If 1 strategy was associated with the most QALYs and lowest costs compared with all other strategies, we classified it as *optimal*. In our cost-effectiveness evaluation, we considered 3 recommended willingness-to-pay thresholds of $50 000/QALY, $100 000/QALY, and $150 000/QALY.^39,40^

#### Subgroup Analysis

We also performed 2 subgroup analyses that evaluated cost-effectiveness of treatment strategies in patients stratified by ER status of their cancer (ie, ER-positive or ER-negative cancers). In these subgroup analyses, we used ER-specific rates of pCR for each neoadjuvant treatment regimen,^5,41^ and for patients with various amounts of residual disease, we used probabilities of distant recurrence as reported in the KATHERINE trial (eTable 1 in the Supplement).^8^ Relative risk of distant recurrence for adjuvant T-DM1 after recurrent disease reported in the KATHERINE trial was lower for ER-positive cancers (RR, 0.48; 95% CI, 0.38-0.67) and ER-negative cancers (RR, 0.50; 95% CI, 0.33-0.74) compared with the total population (RR, 0.60; 95% CI = 0.45-0.79).^8^ Consequently, the results of these subgroups analyses and the base-case results may not be directly comparable.

#### Uncertainty Analysis

We assigned a probability distribution to each input parameter and conducted a probabilistic analysis, also known as *probabilistic sensitivity analysis*, with 1000 iterations to propagate parameter uncertainty to the model output. We evaluated the probability that a given strategy was cost effective using cost-effectiveness acceptability curves and the probability that the strategy associated with the highest net benefit was cost effective using a cost-effectiveness acceptability frontier for willingness-to-pay thresholds from $0 to $200 000/QALY. Furthermore, we examined uncertainty in the results of our model with 1-way sensitivity analyses varying base-case values of the influential parameters (1 at a time) by increases and decreases of up to 30%. These parameters included rates of pCR after each neoadjuvant treatment, distant recurrence risk in patients with residual disease receiving different adjuvant treatments, probability of CHF and acute myeloid leukemia (ie, chemotherapy toxic effects), and costs of distant recurrence health state. Additionally, we conducted a scenario analysis that added adjuvant P to adjuvant H in patients with pCR. We performed all analyses using R statistical software version 3.6.2 (R Project for Statistical Computing).^13^ Data analyses were performed from March 2019 to August 2020.

## Results

### Base-Cases Analysis

Strategy 3 was associated with the highest health benefits (10.73 QALYs) at the lowest costs ($415 833) compared with all other strategies. This strategy dominated all other treatment strategies and was deemed the optimal strategy (Table 2). All other treatment strategies were considered cost-ineffective (eFigure 1 in the Supplement). Strategy 5 was associated with the next highest health benefits, of 10.66 QALYs, and strategy 4 was associated with the third highest health benefits, of 10.31 QALYs. However, these treatment strategies were associated with increased costs (strategy 5: $489 449 and strategy 4: $518 859) compared with strategy 3. Strategy 1 (ie, KATHERINE trial control arm) was associated with the least health benefits (9.67 QALYs) and the third lowest costs ($479 226). Strategy 2 (ie, KATHERINE experimental arm) was associated with the second lowest health benefits (10.22 QALYs) and the second lowest costs ($452 034).

**Table 2.** Cost-effectiveness Results for Base-Case Analysis and Subgroup Analyses

Strategy	Costs, $	QALYs	Incremental		ICER ($/QALY)^a^
			Costs, $	QALYs	
**Base-case analysis**					
Strategy 3^b^	415 833	10.73	NA	NA	Optimal strategy^c^
Strategy 2^d^	452 034	10.22	36 201	−0.51	Dominated
Strategy 1^e^	479 226	9.67	63 393	−1.06	Dominated
Strategy 5^f^	489 449	10.66	73 616	−0.07	Dominated
Strategy 4^g^	518 859	10.31	103 026	−0.42	Dominated
**Subgroup analysis: ER-positive status**					
Strategy 3^b^	433 411	10.59	NA	NA	Cost-effective strategy
Strategy 2^d^	443 837	10.31	10 426	−0.28	Dominated
Strategy 5^f^	485 311	10.73	51 900	0.14	370 714^h^
Strategy 1^e^	490 409	9.53	5098	−1.20	Dominated
Strategy 4^g^	524 681	10.34	39 370	−0.39	Dominated
**Subgroup analysis: ER-negative status**					
Strategy 3^b^	382 103	11.02	NA	NA	Cost-effective strategy
Strategy 2^d^	402 702	10.62	20 599	−0.40	Dominated
Strategy 1^e^	420 985	10.31	38 882	−0.71	Dominated
Strategy 5^f^	443 039	11.09	60 936	0.07	870 514^h^
Strategy 4^g^	482 268	10.59	39 229	−0.50	Dominated

Abbreviations: ER, estrogen receptor; ICER, incremental cost-effectiveness ratio; NA, not applicable; QALY, quality-adjusted life-year.

^a^ Definitions of ICER, dominated status, and willingness-to-pay thresholds included in Methods.

^b^ Neoadjuvant paclitaxel, trastuzumab, and pertuzumab triplet followed by adjuvant dose-dense anthracycline/cyclophosphamide plus trastuzumab emtansine for patients with residual disease and by adjuvant trastuzumab for patients with pathologic complete response.

^c^ The treatment regimen called the *optimal strategy* is a so-called dominant strategy, which leads to the highest health benefits (ie, greatest QALYs) at least costs across all considered treatment regimens.

^d^ Neoadjuvant dose-dense anthracycline/cyclophosphamide followed by paclitaxel, trastuzumab, and pertuzumab followed by adjuvant trastuzumab emtansine for patients with residual disease and followed by adjuvant trastuzumab for patients with pathologic complete response.

^e^ Neoadjuvant dose-dense anthracycline/cyclophosphamide followed by paclitaxel, trastuzumab, and pertuzumab followed by adjuvant trastuzumab for patients with residual disease and for patients with pathologic complete response.

^f^ Neoadjuvant docetaxel, carboplatin, trastuzumab, and pertuzumab followed by adjuvant trastuzumab emtansine for patients with residual disease and followed by adjuvant trastuzumab for patients with partial complete response.

^g^ Neoadjuvant trastuzumab and pertuzumab followed by adjuvant dose-dense anthracycline/cyclophosphamide followed by paclitaxel, trastuzumab, and pertuzumab plus trastuzumab emtansine for patients with residual disease and followed by adjuvant trastuzumab for patients with pathologic complete response.

^h^ The ICER exceeds the willingness-to-pay thresholds of $50 000/QALY, $100 000/QALY, and $150 000/QALY considered in the present study.

### Subgroup Analyses

In patients with ER-positive cancer, strategy 3 was associated with 10.59 QALYs and the least costs, at $433 411, and represented a cost-effective strategy at the 3 willingness-to-pay thresholds (Table 2). In these patients, strategy 5 was associated with the highest health benefits, at 10.73 QALYs; however, this treatment regimen was also associated with increased costs (incremental cost, $51 900) and an ICER of $370 714/QALY. Similarly, in patients with ER-negative cancer, strategy 3 was the cost-effective treatment regimen at the 3 willingness-to-pay thresholds considered, with the least costs, at $382 103, and health benefits of 11.02 QALYs (Table 2). Strategy 5 was associated with the highest health benefits, at 11.09 QALYs, and increased costs (incremental cost, $60 936) and an ICER of $870 514/QALY. Using the 3 willingness-to-pay thresholds, strategy 3 was cost-effective for patients with ER-positive cancer or ER-negative cancer.

### Uncertainty Analyses

**Figure 3.**
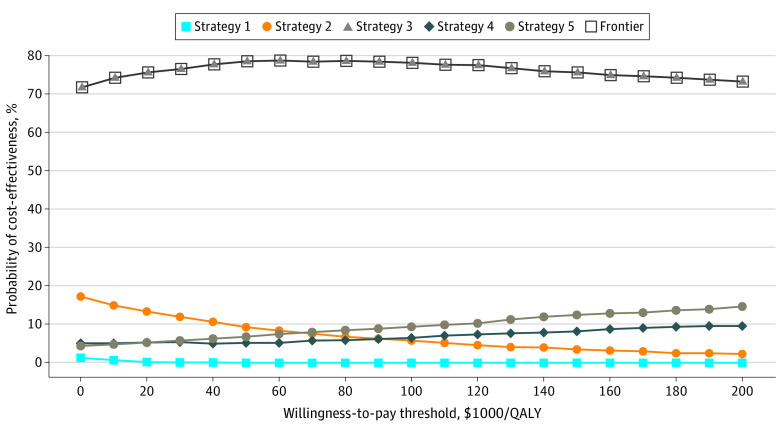
Cost-effectiveness Acceptability Curves and Frontier Frontier indicates the cost-effectiveness acceptability frontier used to evaluate the probability that the strategy with the highest net benefit is cost effective; strategy 1, neoadjuvant dose-dense anthracycline/cyclophosphamide followed by paclitaxel, trastuzumab, and pertuzumab followed by adjuvant trastuzumab for patients with residual disease and for patients with pathologic complete response; strategy 2, neoadjuvant dose-dense anthracycline/cyclophosphamide followed by paclitaxel, trastuzumab, and pertuzumab followed by adjuvant trastuzumab emtansine for patients with residual disease and followed by adjuvant trastuzumab for patients with pathologic complete response; strategy 3, neoadjuvant paclitaxel, trastuzumab, and pertuzumab triplet followed by adjuvant dose-dense anthracycline/cyclophosphamide plus trastuzumab emtansine for patients with residual disease and by adjuvant trastuzumab for patients with pathologic complete response; strategy 4, neoadjuvant trastuzumab and pertuzumab followed by adjuvant dose-dense anthracycline/cyclophosphamide followed by paclitaxel, trastuzumab, and pertuzumab plus trastuzumab emtansine for patients with residual disease and followed by adjuvant trastuzumab for patients with pathologic complete response; strategy 5, neoadjuvant docetaxel, carboplatin, trastuzumab, and pertuzumab followed by adjuvant trastuzumab emtansine for patients with residual disease and followed by adjuvant trastuzumab for patients with partial complete response; and QALY, quality-adjusted life year.

In probabilistic analysis, strategy 3 was associated with the highest probability of cost-effectiveness compared with other strategies (>70% in base-case analysis and >50%-60% in subgroup analyses) and was associated with the highest net monetary benefit across all willingness-to-pay thresholds, from $0 to 200 000/QALY (Figure 3 and eFigure 2 in the Supplement). These findings persisted after changing a number of assumptions. Specifically, strategy 3 continued dominating other strategies as the median age increased to 64 years. We varied the proportion of patients with pCR after neoadjuvant THP (from 32% to 59%), the HRs for distant recurrence after adjuvant DDAC plus T-DM1 (from 0.28 to 0.55), and the costs of distant-recurrence health state (from $97 434 to $180 948). In these sensitivity analyses, strategy 3 remained associated with the highest health benefits and lowest costs (ie, was the optimal strategy) or was the cost-effective strategy across all considered parameter values using the 3 willingness-to-pay thresholds (eFigure 3 in the Supplement). In sensitivity analyses for other influential parameters (performed by increasing and decreasing parameters’ values by up to 30%), strategy 3 remained associated with the highest health benefits and lowest costs or was cost-effective across all considered parameter values using the 3 willingness-to-pay thresholds (eTable 2 in the Supplement). Finally, strategy 3 remined the optimal treatment in scenario analysis assuming adjuvant HP for patients with pCR (eTable 3 in the Supplement).

## Discussion

We performed a model-based economic evaluation that examined the cost-effectiveness of 5 neoadjuvant followed by adjuvant treatment strategies for *ERBB2*-positive breast cancer by modeling our patient population and outcomes based on the KATHERINE trial. This economic evaluation found that neoadjuvant THP followed by adjuvant DDAC and T-DM1 for individuals with residual disease and followed by adjuvant H for individuals with pCR (ie, strategy 3) was the optimal strategy as it was associated with the greatest health benefits and lowest costs compared with other considered treatment strategies. In our results stratified by ER status, strategy 3 was the cost-effective strategy for treating patients with ER-positive or ER-negative cancers using the 3 willingness-to-pay thresholds. In sensitivity analyses that varied pCR rate and recurrence risks with residual disease, strategy 3 remained the optimal strategy or was the cost-effective strategy.

Pathologic complete response is associated with long-term metastasis-free survival, and maximizing rates of pCR was an important goal of clinical trials in the past 20 years. This has been associated with the development of longer, more toxic, and more costly multidrug regimens for *ERBB2*-positive cancers. A crucial recent development was the recognition that adjuvant chemotherapy can improve the survival of patients who do not achieve pCR.^2,8,9^ There is also mounting evidence that pCR is associated with similarly good survival regardless of what chemotherapy regimen is administered.^3,4,11,42,43^ These observations open the opportunity for de-escalation of neoadjuvant chemotherapy and the use of the presence or absence of residual disease to guide subsequent postoperative adjuvant chemotherapy. Starting with a shorter, less toxic, and less expensive neoadjuvant regimen may allow a proportion of patients (20%-40% depending on regimen) who achieve pCR to avoid longer, more toxic regimens, whereas patients with residual disease may be able to receive the remaining part of the most effective current regimens postoperatively as adjuvant therapy.

Providing patients with neoadjuvant treatment associated with decreased rates of pCR (eg, THP vs TCHP) may be associated with decreased neoadjuvant treatment costs. However, it may also be associated with increased adjuvant treatment costs due to patients with residual disease receiving more costly adjuvant treatment when they would have achieved pCR with TCHP. Our model estimates total costs associated with each neoadjuvant-adjuvant treatment strategy, accounting for neoadjuvant and adjuvant treatment costs and simulated health states. We also realize that selecting a treatment strategy involves personal trade-offs. For example, neoadjuvant THP spares many patients (approximately 46%) from receiving adjuvant chemotherapy, but an estimated 7% to 11% of patients with residual disease after THP will receive more chemotherapy (ie, T-DM1) than they would have received if they started by receiving neoadjuvant TCHP or DDAC-THP. These are the 7% to 11% of patients who would have had pCR with the more aggressive initial neoadjuvant therapy.^5,8,17^

To our knowledge, this is the first study to examine the cost-effectiveness of different combinations of neoadjuvant followed by adjuvant treatment strategies for women with *ERBB2*-positive breast cancer. A 2020 study^44^ evaluated the cost-effectiveness of breast cancer treatments but focused on the neoadjuvant setting. That study’s results should not be directly compared with ours, because the treatment strategies differed between the 2 studies. However, we compared the cost-effectiveness results for the treatment strategies that were similar in the 2 studies, and our results were in line with the previous results, indicating that THP represented the preferred neoadjuvant treatment.^44^ Unlike the previous study, our study provided results for various neoadjuvant-adjuvant treatment combinations, where the adjuvant treatment was chosen depending on the provided neoadjuvant treatment.

### Limitations

This study has several limitations. Owing to lack of outcome data from clinical trials, we did not consider additional clinically plausible adjuvant treatment regimens. For example, adjuvant pertuzumab added to trastuzumab increased the 3-year invasive disease-free survival rate from 91% to 93% (*P* = .045) compared with trastuzumab alone.^45^ However, to our knowledge, there are no studies on how adjuvant pertuzumab added to trastuzumab may change the distant recurrence risk in patients with residual disease after neoadjuvant therapy, including neoadjuvant regimens with pertuzumab. Similarly, the ExteNET (Extended Adjuvant Treatment of Breast Cancer With Neratinib) trial^46^ showed that adjuvant neratinib given after adjuvant trastuzumab improved invasive disease-free survival, from 88% to 90% (*P* = .008), compared with placebo, but there are no studies, to our knowledge, on how adjuvant neratinib may alter outcomes in patients with residual disease after neoadjuvant, *ERBB2*-targeted therapy. It is possible that adjuvant pertuzumab and neratinib may improve prognosis among patients with residual disease regardless of T-DM1 administration, but the magnitude of this improvement, if any, is unknown. Our sensitivity analysis suggested that our results may hold up in patient populations with broad ranges of recurrence risk.

There are no randomized clinical trial data, to our knowledge, demonstrating that neoadjuvant THP followed by adjuvant DDAC and T-DM1 for patients with residual disease results in the same long-term outcomes as neoadjuvant THP-DDAC followed by T-DM1 for patients with residual disease, which is a fundamental premise behind our model. Nevertheless, we believe that this is a reasonable assumption based on the National Surgical Adjuvant Breast and Bowel Project Protocol B-27 (NSABP-B-27) trial,^47^ which compared neoadjuvant anthracycline/cyclophosphamide (AC) followed by adjuvant docetaxel with neoadjuvant AC plus docetaxel, and the long-term survival was the same in both docetaxel-containing arms, regardless of administration sequence (as expected, rate of pCR was higher with neoadjuvant AC plus docetaxel compared with AC alone). A clinical trial (A011801 CompassHER2-RD, NCT04457596),^48^ with estimated start date of January 2021, will prospectively test de-escalation strategies using THP as neoadjuvant therapy and reserving further treatment for individuals with residual disease.

## Conclusions

These findings suggest that, in a patient population with *ERBB2*-positive (also known as *HER2*-positive) cancer, like the KATHERINE trial population, neoadjuvant THP followed by adjuvant DDAC and T-DM1 for patients with residual disease and followed by H for patients with pCR is associated with the highest health benefits and lowest costs compared with other treatment regimens. This treatment regimen seems to represent the preferred strategy in ER-positive and ER-negative cancers.

## Notes

Supplement. **eTable 1.** Input Parameters Used in the Decision-Analytic Model **eTable 2.** One-Way Sensitivity Analyses Examining Cost-Effectiveness Results When Varying Values of Influential Input Parameters **eTable 3.** Cost-Effectiveness Results for Scenario Analysis With Adjuvant HP for Pathologic Complete Response **eFigure 1.** Cost-Effectiveness Plane **eFigure 2.** Subgroup Analysis: Cost-Effectiveness Acceptability Curves and Frontier for Patients With ER-Positive and ER-Negative Cancer **eFigure 3.** One-Way Sensitivity Analyses Examining the Cost-Effectiveness of Strategy 3 Using Willingness to Pay of $50 000/QALY, $100 000/QALY, and $150 000/QALY **eReferences**
