# Supplementary material for: Cost-Effectiveness of Neoadjuvant-Adjuvant Treatment Strategies for Women With ERBB2 (HER2)–Positive Breast Cancer
Source: JAMA Netw Open. 2020 Nov 23;3(11):e2027074. doi: 10.1001/jamanetworkopen.2020.27074 (PMC7684449; doi:10.1001/jamanetworkopen.2020.27074)
Supplement: Supplement. — eTable 1. Input Parameters Used in the Decision-Analytic Model eTable 2. One-Way Sensitivity Analyses Examining Cost-Effectiveness Results When Varying Values of Influential Input Parameters eTable 3. Cost-Effectiveness Results for Scenario Analysis With Adjuvant HP for Pathologic Complete Response eFigure 1. Cost-Effectiveness Plane eFigure 2. Subgroup Analysis: Cost-Effectiveness Acceptability Curves and Frontier for Patients With ER-Positive and ER-Negative Cancer eFigure 3. One-Way Sensitivity Analyses Examining the Cost-Effectiveness of Strategy 3 Using Willingness to Pay of $50 000/QALY, $100 000/QALY, and $150 000/QALY eReferences [file jamanetwopen-e2027074-s001.pdf]

Supplemental Online Content

Kunst N, Wang SY, Hood A, et al. Cost-effectiveness of neoadjuvant-adjuvant treatment strategies for women with *ERBB2* (*HER2*)–positive breast cancer. *JAMA Netw Open*. 2020;3(11):e2027074. doi:10.1001/jamanetworkopen.2020.27074

eTable 1. Input Parameters Used in the Decision-Analytic Model

eTable 2. One-Way Sensitivity Analyses Examining Cost-Effectiveness Results When Varying Values of Influential Input Parameters

eTable 3. Cost-Effectiveness Results for Scenario Analysis With Adjuvant HP for Pathologic Complete Response

eFigure 1. Cost-Effectiveness Plane

eFigure 2. Subgroup Analysis: Cost-Effectiveness Acceptability Curves and Frontier for Patients With ER-Positive and ER-Negative Cancer

eFigure 3. One-Way Sensitivity Analyses Examining the Cost-Effectiveness of Strategy 3 Using Willingness To Pay of $50 000/QALY, $100 000/QALY, and $150 000/QALY

eReferences

This supplemental material has been provided by the authors to give readers additional information about their work.

© 2020 Kunst N et al. *JAMA Network Open.*

eTable 1. Input Parameters Used in the Decision-Analytic Model

Input parameters	Value	PA distribution
**Subgroup analysis: ER-positive subgroup**		
Proportion of patients with pCR after neoadjuvant treatment		
HP	5.9%[e1]	Beta(α=3, β=48)
THP	26%[e1]	Beta(α=13, β=37)
ddAC/THP	52.2%[e2]*	Beta(α=12, β=11)
TCHP	43.8%[e3]	Beta(α=56, β=72)
Distant recurrence		
3-year distant recurrence probability with H after RD (reference group)	15.9%[e4]	Beta(α= 118, β=625)
Relative risk of distant recurrence		
RR of DR for T-DM1 after RD	0.48[e4]	Log normal (μ= -0.73, σ=0.08)
RR of DR for ddAC/THP followed by T-DM1 after RD	0.38[e4,5]†	Truncated normal (a=0.18, b=0.48)
RR of DR for ddAC followed by T-DM1 after RD	0.33[e4,5]†	Truncated normal (a=0.182, b=0.48)
RR of DR for H after pCR	0.18[e6,7]	Log normal (μ= -1.7, σ=0.33)
**Subgroup analysis: ER-negative subgroup**		
Proportion of patients with pCR after neoadjuvant treatment		
HP	27.3%[e1]	Beta(α=15, β=40)
THP	63.2%[e1]	Beta(α=36, β=21)
ddAC/THP	80%[e8]	Beta(α=20, β=5)
TCHP	73.2%[e3]	Beta(α=60, β=22)
Distant recurrence		
3-year distant recurrence probability with H after RD (reference group)	15.9%[e4]	Beta(α= 104, β=540)
Relative risk of distant recurrence		
RR of DR for T-DM1 after RD	0.50[e4]	Log normal (μ=-0.69, σ=0.11)
RR of DR for ddAC/THP followed by T-DM1 after RD	0.36[e4,5]†	Truncated normal (a=0.18, b=0.50)
RR of DR for ddAC followed by T-DM1 after RD	0.27[e4,5]†	Truncated normal (a=0.18, b=0.50)
RR of DR for H after pCR	0.18[e6,7]	Log normal(μ=-1.70, σ=0.18)
**Scenario analysis: adjuvant HP for pCR**		
3-year distant recurrence probability with H after RD (reference group)	15.9%[e4]	Beta(α= 118, β=625)
RR of DR for H after pCR	0.12[e5]‡	Log normal (μ=-2.13, σ=0.21)
**Costs**[§]		
**Costs of neoadjuvant treatment regimens**		
**Neoadjuvant treatment regimen: HP**		
Treatment		
Trastuzumab 8mg/kg load, then 6mg/kg x 3	$33,087.65[e9]	
Pertuzumab 840mg load, then 420mg x 3	$31,301.34[e9]	
Total HP cost	$64,388.99[e9]	Gamma (α=25, β=2575.56)
**Neoadjuvant treatment regimen: THP**		
Premedication		

© 2020 Kunst N et al. *JAMA Network Open.*

Dexamethasone 10mg IV x 2	$21.88[e9]	
Diphenhydramine 25mg PO x 2	$0.08[e9]	
Famotidine 20mg PO x 2	$2.62[e9]	
Dexamethasone 8mg PO x 10	$24.87[e9]	
Treatment		
Trastuzumab 8mg/kg load, then 6mg/kg x 3	$33,087.65[e9]	
Pertuzumab 840mg load, then 420mg x 3	$31,301.34[e9]	
Weekly paclitaxel 80mg/m2 x 12	$989.14[e9]	
Total THP cost	$65,427.58[e9]	Gamma (α= 25, β=2617.10)
**Neoadjuvant treatment regimen: ddAC/THP**		
Premedication paclitaxel		
Dexamethasone 10mg IV x 2	$21.88[e9]	
Diphenhydramine 25mg PO x 2	$0.08[e9]	
Famotidine 20mg PO x 2	$2.62[e9]	
Dexamethasone 8mg PO x 10	$24.87[e9]	
Premedication ddAC		
Palonosetron 0.25mg IVP x 4	$1,199.58[e9]	
Dexamethasone 12mg PO x 4	$15.32[e9]	
Aprepitant 130mg IV x 4	$1,642.58[e9]	
Treatment		
Trastuzumab 8mg/kg load, then 6mg/kg x 3	$33,087.65[e9]	
Pertuzumab 840mg load, then 420mg x 3	$31,301.34[e9]	
Weekly paclitaxel 80mg/m2 x 12	$989.14[e9]	
Doxorubicin 60mg/m2 x 4	$2,465.61[e9]	
Cyclophosphamide 600mg/m2 x 4	$4,878.54[e9]	
Supportive care		
Pegfilgrastim 6mg SQ x 4	$31,128.29[e9]	
Take-home medications		
Dexamethasone 8mg x 3 days x 4 cycles	$29.77[e9]	
Total ddAC/THP cost	$106,787.35[e9]	Gamma (α=25, β=4271.49)
**Neoadjuvant treatment regimen: TCHP**		
Premedication TCHP		
Dexamethasone 10mg IV x 2	$21.88[e9]	
Dexamethasone 12mg PO x 4	$15.32[e9]	
Aprepitant 130mg IV x 6	$2,463.86[e9]	
Palonosetron 0.25mg IVP x 6	$1,799.38[e9]	
Treatment		
Docetaxel 75mg/m2 x 6	$8,894.75[e9]	
Carboplatin AUC 6 x 6	$859.22[e9]	
Trastuzumab 8mg/kg load, then 6mg/kg x 5	$48,658.31[e9]	
Pertuzumab 840mg load, then 420mg x 5	$43,821.87[e9]	
Supportive care		
Pegfilgrastim 6mg SQ x 6	$46,692.43[e9]	
Take-home medications		
Dexamethasone 8mg x 2 days x 6 cycles	$29.85[e9]	
Total TCHP cost	$153,256.87[e9]	Gamma (α=25, β=6130.28)
**Costs of adjuvant treatment regimens[e9]**		
**Adjuvant treatment regimen: H**		
Treatment		

© 2020 Kunst N et al. *JAMA Network Open.*

Trastuzumab 6mg/kg x 14	$108,994.61[e9]	
Total H cost	$108,994.61[e9]	Gamma (α=25, β=4359.78)
**Adjuvant treatment regimen: HP (scenario analysis)**		
Treatment		
Trastuzumab 6mg/kg x 14	$108,994.61[e9]	
Pertuzumab 420mg x 14	$87,643.74[e9]	
Total HP cost	$196,638.35[e9]	Gamma (α=25, β= 7865.53)
**Adjuvant treatment regimen: T-DM1**		
Premedication		
Ondansetron 16mg ODT premedication x 14	$1,141.32[e9]	
Treatment		
T-DM1 x 14	$156,729.22[e9]	
Total T-DM1 cost	$157,870.55[e9]	Gamma (α=25, β=6314.82)
**Adjuvant treatment regimen: ddAC/THP followed by T-DM1**		
Treatment		
Total ddAC/THP	$106,787.35[e9]	
Total T-DM1 cost	$157,870.55[e9]	
Total ddAC/THP followed by T-DM1 cost	$264,657.90[e9]	Gamma (α=25, β=10586.32)
**Adjuvant treatment regimen: ddAC followed by T-DM1**		
Treatment with ddAC		
Doxorubicin 60mg/m2 x 4	$2,465.61[e9]	
Cyclophosphamide 600mg/m2 x 4	$4,878.54[e9]	
Premedication for ddAC		
Palonosetron 0.25mg IVP x 4	$1,199.58[e9]	
Dexamethasone 12mg PO x 4	$15.32[e9]	
Aprepitant 130mg IV x 4	$1,642.58[e9]	
Supportive care for ddAC		
Pegfilgrastim 6mg SQ x 4	$31,128.29[e9]	
Take-home medications for ddAC		
Dexamethasone 8mg x 3 days x 4 cycles	$29.85[e9]	
Total ddAC cost	$41,359.77[e9]	
Total T-DM1 cost	$157,870.55[e9]	
Total ddAC followed by T-DM1 cost	$199,230.31[e9]	Gamma (α=25, β=7969.21)
**Adjuvant treatment regimen: Adjuvant H after neoadjuvant TCHP**		
Treatment		
Trastuzumab 6mg/kg x 12	$93,423.95[e9]	
Total cost adjuvant H after neoadjuvant TCHP	$93,423.95[e9]	Gamma (α=25, β=3736.96)
**Adjuvant treatment regimen T-DM1 after neoadjuvant TCHP**		
Treatment		
T-DM1 x 12	$134,339.33[e9]	

© 2020 Kunst N et al. *JAMA Network Open.*

Premedication		
Ondansetron 16mg ODT premedication x 12	$978.28[e9]	
Total cost of adjuvant T-DM1 after neoadjuvant TCHP	$135,317.61[e9]	Gamma (α=25, β=5412.70)

Abbreviations: ddAC, dose dense anthracycline/cyclophosphamide; ddAC/THP, dose dense anthracycline/cyclophosphamide followed by paclitaxel, trastuzumab and pertuzumab; DR, distant recurrence; H, trastuzumab; HP, trastuzumab and pertuzumab; HR, hazard ratio; LR, local recurrence; pCR, pathologic complete response; RD, residual disease; RR, relative risk; TCHP, docetaxel, carboplatin, trastuzumab and pertuzumab; T-DM1, trastuzumab emtansine; THP, paclitaxel, trastuzumab and pertuzumab triplet.
*The proportion of patients with pCR after ddAC/THP was taken from Buzdar, et al.[e2] Because the reported estimate was considered an overestimation of real-world clinical practice, we adjusted this estimated based on clinical expert opinion.
†This is an assumption because of no evidence for this setting. We assumed that the true value was between a 5-year probability of distant recurrence of 5% in patients with pCR receiving H from Symmans, et al.[e5] for the proportion of patients that would be categorized to pCR if treated with neoadjuvant ddAC/THP, and a 3-year probability of distant recurrence for the remaining patients with RD receiving T-DM1 from von Minckwitz, et al. [e4]
‡To estimate RR for adjuvant HP, we used 3-year invasive disease-free survival with pCR from Symmans, et al.[e5] but assumed that this survival would not change until year 5. This is because disease-free survival with pCR for adjuvant H was provided at year 5 and 10 and showed that the survival did not change in patients with pCR between year 5 and year 10. Using a 3-year disease-free survival for HP would likely overestimate the 1-year probabilty of recurrence with adjuvant HP, i.e., the 1-year probability of recurrence with H would be lower than the 1-year probabilty of recurrence with HP.
§All costs are expressed in USD 2020. Where necessary, we inflated unit costs to USD 2020 using Consumer Price Index (CPI)[e10]

eTable 2. One-Way Sensitivity Analyses Examining Cost-Effectiveness Results When Varying Values of Influential Input Parameters

Parameter type	Cost-effectiveness results*†
**Proportion of patients with pCR**	
Neoadjuvant HP	
11%	**Strategy 3**: Dominant
15%	**Strategy 3**: Dominant
19%	**Strategy 3**: Dominant
22%	**Strategy 3**: Dominant
25%	**Strategy 3**: Dominant
29%	**Strategy 3**: Dominant
Neoadjuvant ddAC/THP	
40%	**Strategy 3**: Dominant
46%	**Strategy 3**: Dominant
53%	**Strategy 3**: Dominant
60%	**Strategy 3**: Dominant
67%	**Strategy 3**: Dominant
74%	**Strategy 3**: Dominant
Neoadjuvant TCHP	
37%	**Strategy 3**: Dominant
43%	**Strategy 3**: Dominant
49%	**Strategy 3**: Dominant
56%	**Strategy 3**: Dominant
62%	**WTP of $50,000/QALY $100,000/QALY, and $150,000/QALY: Strategy 3**: Cost effective
68%	**WTP of $50,000/QALY $100,000/QALY, and $150,000/QALY: Strategy 3**: Cost effective
**HR for distant recurrence**	
T-DM1	
0.42	**WTP of $50,000/QALY $100,000/QALY, and $150,000/QALY: Strategy 3**: Cost effective
0.49	**WTP of $50,000/QALY $100,000/QALY, and $150,000/QALY: Strategy 3**: Cost effective
0.56	**WTP of $50,000/QALY $100,000/QALY, and $150,000/QALY: Strategy 3**: Cost effective
0.64	**Strategy 3**: Dominant
0.71	**Strategy 3**: Dominant
0.78	**Strategy 3**: Dominant
ddAC/THP followed by T-DM1	
0.36	**Strategy 3**: Dominant
0.42	**Strategy 3**: Dominant
0.49	**Strategy 3**: Dominant
0.55	**Strategy 3**: Dominant
0.61	**Strategy 3**: Dominant
0.67	**Strategy 3**: Dominant
**Chemotherapy toxicity**	
**1-year probability of CHF in patients with non-AC chemotherapy**	
2.59%	**Strategy 3**: Dominant
4.81%	**Strategy 3**: Dominant
**1-year probability of AML in patients with no chemotherapy**	
0.049%	**Strategy 3**: Dominant
0.091%	**Strategy 3**: Dominant

© 2020 Kunst N et al. *JAMA Network Open.*

Strategy 1: neoadjuvant ddAC/THP and adjuvant H for RD; H for pCR. Strategy 2: neoadjuvant ddAC/THP and adjuvant T-DM1 for RD; H for pCR. Strategy 3: neoadjuvant THP and adjuvant ddAC+T-DM1 for RD; H for pCR. Strategy 4: neoadjuvant HP and adjuvant ddAC/THP+T-DM1 for RD; H for pCR. Strategy 5: neoadjuvant TCHP and adjuvant T-DM1 for RD; H for pCR.
Abbreviations: ddAC, dose dense anthracycline/cyclophosphamide; ddAC/THP, dose dense anthracycline/cyclophosphamide followed by paclitaxel, trastuzumab and pertuzumab; H, Trastuzumab; HP, trastuzumab and pertuzumab; ICER, incremental cost-effectiveness ratio; pCR, pathologic complete response; QALY, quality-adjusted life year; TCHP, docetaxel, carboplatin, trastuzumab and pertuzumab; T-DM1, trastuzumab emtansine; THP, paclitaxel, trastuzumab and pertuzumab triplet.
*The incremental cost-effectiveness ratio (ICER) presents the incremental costs per incremental quality-adjusted life years (QALYs) relative to the next least costly strategy. Strategies are categorized as dominated if they lead to fewer QALYs at higher costs than the alternative. So-called dominant strategy results in the highest effectiveness (i.e., greatest QALYs) at least costs across all the considered treatment regimens. In the present cost-effectiveness evaluation, we considered three commonly used willingness-to-pay thresholds of $50,000/QALY, $100,000/QALY, and $150,000/QALY.[e11,12]
†All costs are expressed in USD 2020. Where necessary, we inflated unit costs to USD 2020 using Consumer Price Index (CPI)[e10]

© 2020 Kunst N et al. *JAMA Network Open.*

eTable 3. Cost-Effectiveness Results for Scenario Analysis With Adjuvant HP for Pathologic Complete Response

Strategy	Costs	QALYs	Incremental costs	Incremental QALYs	ICER*, $\Delta Costs/\Delta QALYs$
**Base-case analysis**					
**Strategy 3**<br>***Neoadjuvant THP***<br>***Adjuvant ddAC+T-DM1 for RD;***<br>***H for pCR***	**$442,017**	**10.88**	**-**	**-**	**Optimal strategy†**
**Strategy 5**<br>*Neoadjuvant TCHP*<br>*Adjuvant T-DM1 for RD;*<br>*H for pCR*	$473,335	10.84	$31,318	-0.04	Dominated
**Strategy 2**<br>*Neoadjuvant ddAC/THP*<br>*Adjuvant T-DM1 for RD;*<br>*H for pCR*	$485,033	10.39	$43,016	-0.49	Dominated
**Strategy 4**<br>*Neoadjuvant HP*<br>*Adjuvant ddAC/THP+T-DM1 for RD;*<br>*H for pCR*	$528,518	10.36	$86,501	-0.52	Dominated
**Strategy 1**<br>*Neoadjuvant ddAC/THP*<br>*Adjuvant H for RD; H for pCR*	$550,586	9.84	$108,569	-1.04	Dominated

Abbreviations: ddAC, dose dense anthracycline/cyclophosphamide; ddAC/THP, dose dense anthracycline/cyclophosphamide followed by paclitaxel, trastuzumab and pertuzumab; ER, estrogen-receptor status; H, trastuzumab; HP, trastuzumab and pertuzumab; ICER, incremental cost-effectiveness ratio; pCR, pathologic complete response; QALY, quality-adjusted life year; RD, residual disease; TCHP, docetaxel, carboplatin, trastuzumab and pertuzumab; T-DM1, trastuzumab emtansine; THP, paclitaxel, trastuzumab and pertuzumab triplet.

*The incremental cost-effectiveness ratio (ICER) presents the incremental costs per incremental quality-adjusted life years (QALYs) relative to the next least costly strategy. Strategies are categorized as dominated if they lead to fewer QALYs at higher costs than the alternative. In the present cost-effectiveness evaluation, we considered three commonly used willingness-to-pay thresholds of $50,000/QALY, $100,000/QALY, and $150,000/QALY [e11,12].

†The treatment regimen called the optimal strategy is a so-called dominant strategy, which leads to the highest effectiveness (i.e., greatest QALYs) at least costs across all the considered treatment regimens.

© 2020 Kunst N et al. *JAMA Network Open.*

eFigure 1. Cost-Effectiveness Plane

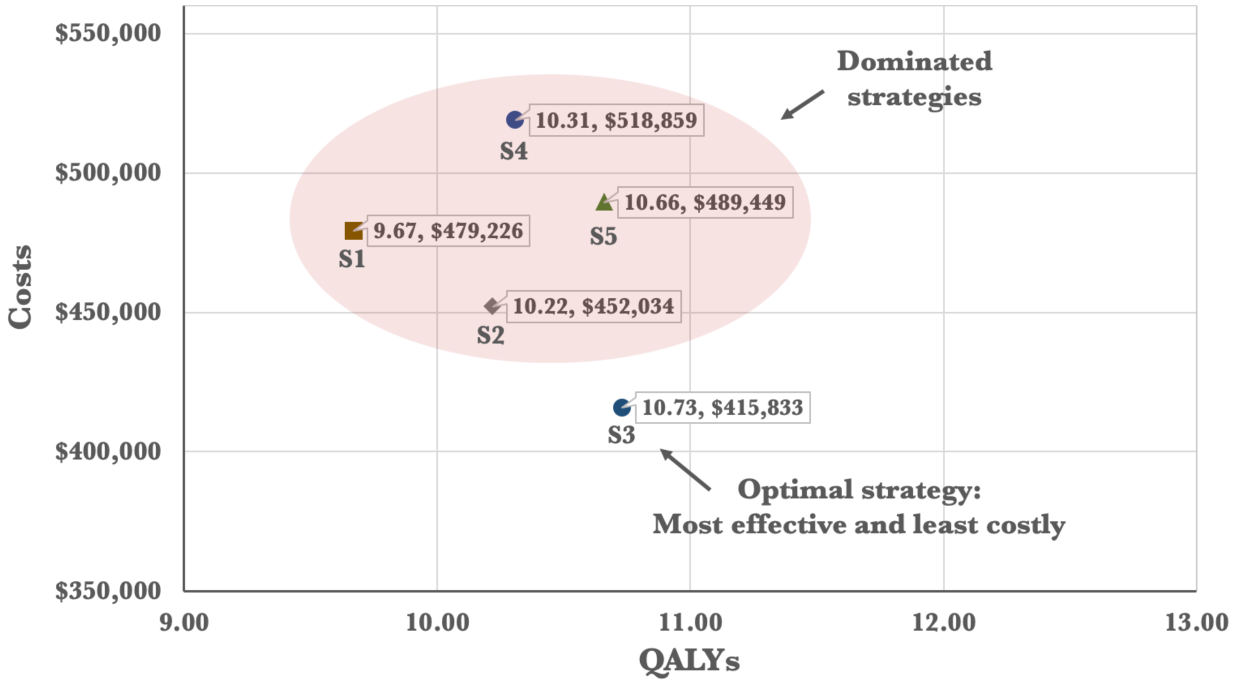

The cost-effectiveness plane visually presents the difference in costs and QALYs between all the considered treatment strategies by plotting the costs on the y-axis against QALYs on the x-axis. The dominated strategies are included in the red circle and the optimal strategy is indicated. Strategy 1: neoadjuvant ddAC/THP and adjuvant H for RD; H for pCR. Strategy 2: neoadjuvant ddAC/THP and adjuvant T-DM1 for RD; H for pCR. Strategy 3: neoadjuvant THP and adjuvant ddAC+T-DM1 for RD; H for pCR. Strategy 4: neoadjuvant HP and adjuvant ddAC/THP+T-DM1 for RD; H for pCR. Strategy 5: neoadjuvant TCHP and adjuvant T-DM1 for RD; H for pCR.
Abbreviations: ddAC, dose dense anthracycline/cyclophosphamide; ddAC/THP, dose dense anthracycline/cyclophosphamide followed by paclitaxel, trastuzumab and pertuzumab; ER, estrogen-receptor status; H, trastuzumab; HP, trastuzumab and pertuzumab; pCR, pathologic complete response; QALY, quality-adjusted life year; TCHP, docetaxel, carboplatin, trastuzumab and pertuzumab; T-DM1, trastuzumab emtansine; THP, paclitaxel, trastuzumab and pertuzumab triplet.

© 2020 Kunst N et al. *JAMA Network Open.*

eFigure 2. Subgroup Analysis: Cost-Effectiveness Acceptability Curves and Frontier for Patients With ER-Positive and ER-Negative Cancer

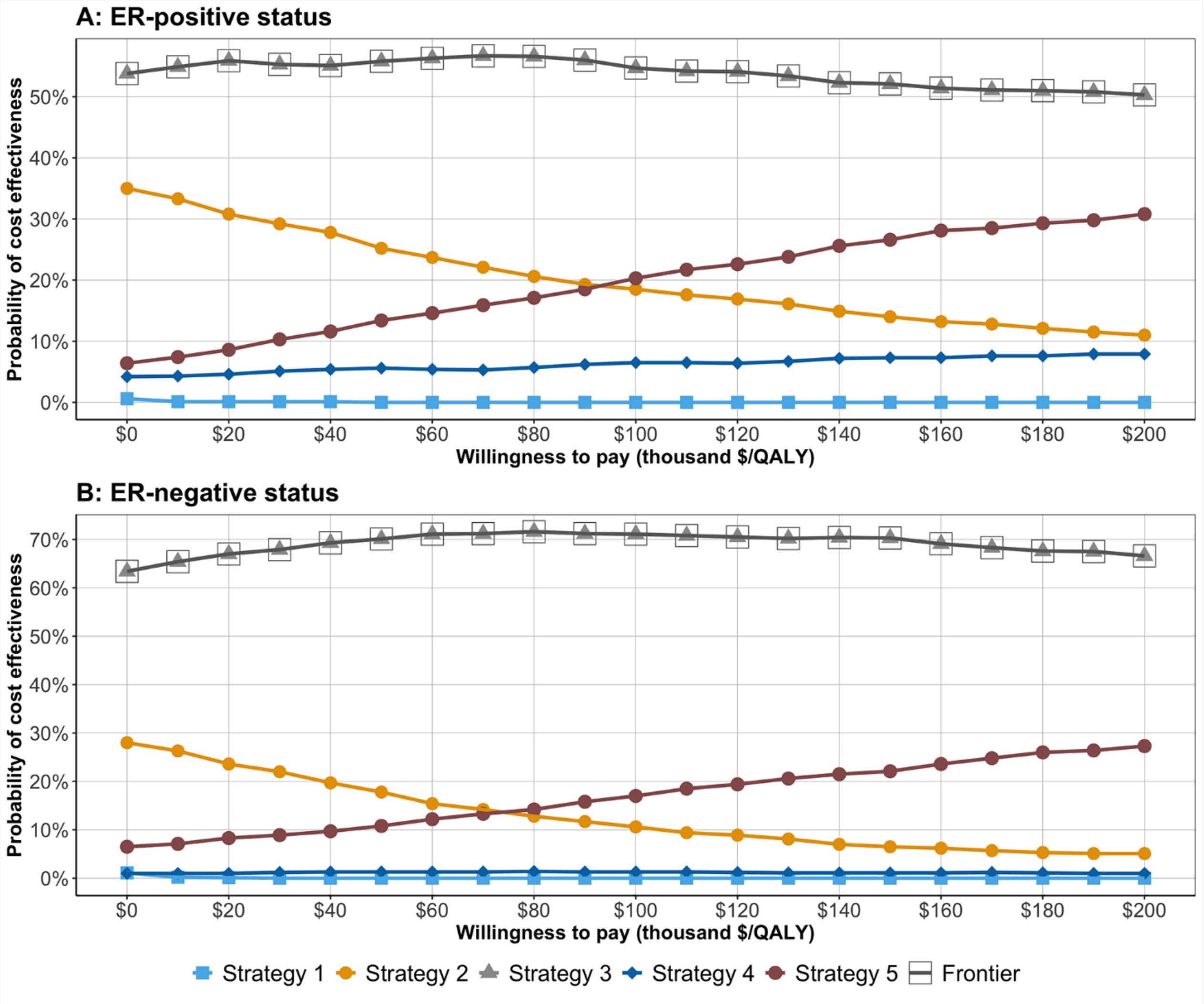

The cost-effectiveness acceptability curves (CEACs) report the probability that a given strategy has the highest net monetary benefit at a willingness-to-pay threshold between $0 and $200,000/QALY. The cost-effectiveness frontier (CEAF) indicates the probability that the strategy with the highest net benefit is cost effective. Strategy 1: neoadjuvant ddAC/THP and adjuvant H for RD; H for pCR. Strategy 2: neoadjuvant ddAC/THP and adjuvant T-DM1 for RD; H for pCR. Strategy 3: neoadjuvant THP and adjuvant ddAC+T-DM1 for RD; H for pCR. Strategy 4: neoadjuvant HP and adjuvant ddAC/THP+T-DM1 for RD; H for pCR. Strategy 5: neoadjuvant TCHP and adjuvant T-DM1 for RD; H for pCR.

Abbreviations: ddAC, dose dense anthracycline/cyclophosphamide; ddAC/THP, dose dense anthracycline/cyclophosphamide followed by paclitaxel, trastuzumab and pertuzumab; ER, estrogen-receptor status; H, Trastuzumab; HP, trastuzumab and pertuzumab; pCR, pathologic complete response; QALY, quality-adjusted life year; TCHP, docetaxel, carboplatin, trastuzumab and pertuzumab; T-DM1, trastuzumab emtansine; THP, paclitaxel, trastuzumab and pertuzumab triplet.

© 2020 Kunst N et al. *JAMA Network Open.*

eFigure 3. One-Way Sensitivity Analyses Examining the Cost-Effectiveness of Strategy 3 Using Willingness To Pay of $50 000/QALY, $100 000/QALY, and $150 000/QALY

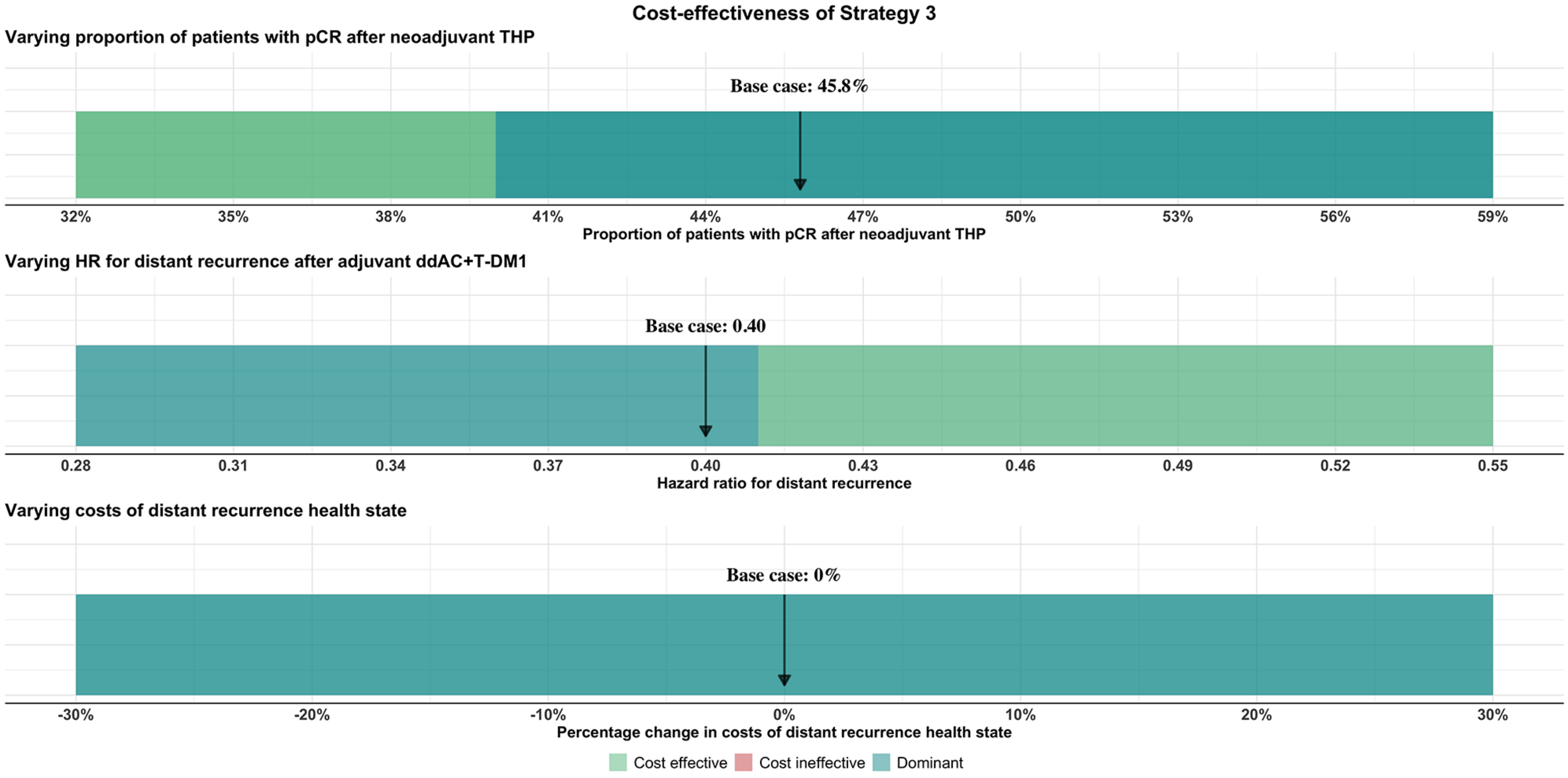

The one-way sensitivity analyses report the cost-effectiveness results for Strategy 3 (i.e., neoadjuvant THP and adjuvant ddAC+T-DM1 for RD and adjuvant H for pCR) at a willingness-to-pay thresholds of $50,000/QALY, $100,000/QALY, and $150,000/QALY when varying +/- 30% the proportion of patients with pCR after neoadjuvant THP, the HR for distant recurrence after adjuvant ddAC+T-DM1, and the costs of distant recurrence health state. Note that the green area indicates that Strategy 3 is cost-effective at the three considered WTP thresholds (i.e., $50,000/QALY, $100,000/QALY, and $150,000/QALY).

Abbreviations: ddAC, dose dense anthracycline/cyclophosphamide; DR, distant recurrence; HR, hazard ratio; QALY, quality-adjusted life year; pCR, pathologic complete response; THP TCHP, paclitaxel, trastuzumab and pertuzumab; T-DM1, trastuzumab emtansine; WTP, willingness to pay.

© 2020 Kunst N et al. *JAMA Network Open.*

eReferences

e1. Gianni L, Pienkowski T, Im YH, et al: Efficacy and safety of neoadjuvant pertuzumab and trastuzumab in women with locally advanced, inflammatory, or early HER2-positive breast cancer (NeoSphere): a randomised multicentre, open-label, phase 2 trial. The Lancet Oncology 13:25-32, 2012

e2. Buzdar AU, Ibrahim NK, Francis D, et al: Significantly higher pathologic complete remission rate after neoadjuvant therapy with trastuzumab, paclitaxel, and epirubicin chemotherapy: results of a randomized trial in human epidermal growth factor receptor 2-positive operable breast cancer. J Clin Oncol 23:3676-85, 2005

e3. Hurvitz SA, Martin M, Symmans WF, et al: Pathologic complete response (pCR) rates after neoadjuvant trastuzumab emtansine (T-DM1 [K]) + pertuzumab (P) vs docetaxel + carboplatin + trastuzumab + P (TCHP) treatment in patients with HER2-positive (HER2+) early breast cancer (EBC) (KRISTINE). Journal of Clinical Oncology 34:500-500, 2016

e4. von Minckwitz G, Huang CS, Mano MS, et al: Trastuzumab emtansine for residual invasive HER2-positive breast cancer. New England Journal of Medicine 380:617-628, 2019

e5. Symmans WF, Wei C, Gould R, et al: Long-term prognostic risk after neoadjuvant chemotherapy associated with residual cancer burden and breast cancer subtype. Journal of Clinical Oncology 35:1049-1060, 2017

e6. Gonzalez-Angulo AM, McGuire SE, Buchholz TA, et al: Factors predictive of distant metastases in patients with breast cancer who have a pathologic complete response after neoadjuvant chemotherapy. Journal of Clinical Oncology 23:7098-104, 2005

e7. Cortazar P, Zhang L, Untch M, et al: Pathological complete response and long-term clinical benefit in breast cancer: the CTNeoBC pooled analysis. Lancet 384:164-72, 2014

e8. Foldi J, Mougalian S, Silber A, et al: Single-arm, neoadjuvant, phase II trial of pertuzumab and trastuzumab administered concomitantly with weekly paclitaxel followed by 5-fluoruracil, epirubicin, and cyclophosphamide (FEC) for stage I-III HER2-positive breast cancer. Breast Cancer Research and Treatment 169:333-340, 2018

e9. Mckesson Connect. Irving, TX: Mckesson Corporation. 2019. https://connect.mckesson.com/portal/site/smo/template.LOGIN/. Accessed February 8, 2019.

e10. U.S. Bureau of Labor Statistics. CPI inflation calculator. https://www.bls.gov/data/inflation_calculator.htm. Accessed July 16, 2018.

e11. Neumann PJ, Cohen JT, Weinstein MC: Updating cost-effectiveness — the curious resilience of the $50,000-per-QALY threshold. New England Journal of Medicine 371:796-797, 2014

e12. Weinstein MC: How much are Americans willing to pay for a quality-adjusted life year? Medical Care 46:343-5, 2008

© 2020 Kunst N et al. *JAMA Network Open.*
